# Chemical Composition, Antioxidant and Antimicrobial Activities of *Thymus capitata* Essential Oil with Its Preservative Effect against *Listeria monocytogenes* Inoculated in Minced Beef Meat

**DOI:** 10.1155/2014/152487

**Published:** 2014-02-25

**Authors:** Nariman El Abed, Belhassen Kaabi, Mohamed Issam Smaali, Meriem Chabbouh, Kamel Habibi, Mondher Mejri, Mohamed Nejib Marzouki, Sami Ben Hadj Ahmed

**Affiliations:** ^1^Laboratory of Protein Engineering and Bioactive Molecules (LIP-MB), Institut National de Sciences Appliquées et de Technologie (INSAT), BP 676, 1080 Tunis Cedex, Tunisia; ^2^Laboratoire d'Epidémiologie et d'Ecologie des Parasites, Institut Pasteur de Tunis, BP 74, 1002 Tunis Belvédère, Tunisia; ^3^Institut National Agronomique de Tunisie, 1002 Tunis, Tunisia; ^4^Institut Supérieur des Etudes Technologiques de Zaghouan, 1121 Mograne, Tunisia

## Abstract

The chemical composition, antioxidant and antimicrobial activities, and the preservative effect of *Thymus capitata *essential oil against *Listeria monocytogenes *inoculated in minced beef meat were evaluated. The essential oil extracted was chemically analyzed by gas chromatography-mass spectrometry. Nineteen components were identified, of which carvacrol represented (88.89%) of the oil. The antioxidant activity was assessed *in vitro *by using both the DPPH and the ABTS assays. The findings showed that the essential oil exhibited high antioxidant activity, which was comparable to the reference standards (BHT and ascorbic acid) with IC_50_ values of 44.16 and 0.463 **μ**g/mL determined by the free-radical scavenging DPPH and ABTS assays, respectively. Furthermore, the essential oil was evaluated for its antimicrobial activity using disc agar diffusion and microdilution methods. The results demonstrated that the zone of inhibition varied from moderate to strong (15–80 mm) and the minimum inhibition concentration values ranged from 0.32 to 20 mg/mL. In addition, essential oil evaluated *in vivo *against *Listeria monocytogenes *showed clear and strong inhibitory effect. The application of 0.25 or 1% (v/w) essential oil of *T. capitata *to minced beef significantly reduced the *L. monocytogenes *population when compared to those of control samples (*P-value  *<0.01).

## 1. Introduction

The problems of spoilage and food poisoning, mainly by oxidation processes or by microorganism activity, during production and storage are still concerns for both the food industry and consumers, despite the use of synthetic chemical additives and various preservation methods [1–3]. However, the side effects of some synthetic antioxidants used in food processing, such as butylated hydroxytoluene (BHT) and butylated hydroxyanisole (BHA), have already been documented. They showed carcinogenic effects in living organisms [4, 5]. Consequently, there has been increasing interest in developing new types of effective and nontoxic natural antioxidant and antimicrobial compounds both to prevent the growth of food- borne and spoiling microbes and to extend the shelf-life of foods [6, 7]. In this context, medicinal and aromatic plants have emerged as an alternative to synthetic products, used not only in traditional medicine but also in a number of food and pharmaceutical products, due to their high content of phenolic compounds, their nutritional properties, and bioactivity [8].

*Thymus capitata* is a Mediterranean herb of the Lamiaceae family that grows mainly in northern Tunisia [9]. This species is an aromatic plant, mostly used (fresh or dried) as a spice, in some Tunisian traditional meat dishes, both for its preservative qualities and its savory taste. In Tunisian folk medicine, *Thymus* species are well known as medicinal plants because of their biological and pharmacological properties, which include antiasthmatic, antiseptic, antimycotic, spasmolytic, anti-inflammatory, antimicrobial and, antioxidant activities [9–12]. Recently, *Thymus* species essential oils (EOs) and their components gained increasing importance because of their wide acceptance by consumers and other exploitations and potential multipurpose functional use [9].

Generally, the essential oils (EOs) are aromatic and volatile liquids extracted from plant materials, such as flowers, roots, bark, leaves, seeds, peel, fruits, wood, and whole plant. They are considered to be plant secondary metabolites, which play an important role in plant defense as they often possess antimicrobial and antioxidant properties [13–15]. For these reasons, EOs have been primarily used, in the food industry, as flavoring agents in food system and can be used as natural antimicrobials in food preservation (extending shelf-life) [15, 16] against a wide range of food spoiling microbes.

Previous phytochemical studies of the genus *Thymus* EOs have reported the presence of a number of bioactive compounds, including carvacrol, thymol, *p-*cymene, and *γ*-terpinene, which have been reported to have many biological activities [3, 9, 11]. Figueiredo et al. in 2008 [11] have demonstrated that EOs of Portuguese *T. capitata* presented great chemical homogeneity characterized by a relatively high amount of carvacrol.

In addition, to our knowledge, there are no published studies that have evaluated the preservative effect of *T. capitata* EO against *L. monocytogenes* in minced meat, the causative agent of listeriosis, one of the most virulent foodborne diseases. Human infection predominantly occurs as a result of occasional contamination of ready-to-eat and raw food products, particularly meat products [17, 18]. Listeriosis has been associated with a mortality rate as high as 30–40% [19]. The ubiquitous prevalence of this pathogen in nature, its ability to proliferate at temperature near 0°C, and its resistance to certain preservatives has resulted in an extensive effort to develop processes to control its growth in foods [20].

Today, different strategies are applied in order to control pathogens in meats, and interest has been focused on the application of EOs as a safe and effective alternative to chemical preservative. Their application in controlling pathogens could reduce the risk of foodborne outbreak and assure consumers safe meat products. The chemical composition and antimicrobial properties of EOs extracted from diverse plant species have been demonstrated using a variety of experimental methods [21, 22].

The purposes of the present work are (i) to evaluate the chemical composition of Tunisian *T. capitata* EOs by GC-MS and compare it to previous published works, (ii) to confirm *in vitro* the antioxidant activity of this EO, and (iii) to assess* in vitro* its antimicrobial activities against a selected group of bacteria strains. Besides, this study was also designed to determine the efficacy of *T. capitata* EO in inhibiting *L. monocytogenes* growth in model minced beef meat during refrigerated storage.

## 2. Materials and Methods

### 2.1. Materials and Chemicals

Chemicals: 2,2-diphenyl-2-picrylhydrazyl hydrate (DPPH), 2,6-di-tert-butyl-4-methylphenol (BHT), 2,2′-azinobis (3-ethylbenzothiazoline-6-sulfonic acid) (ABTS), ascorbic acid, dimethyl sulfoxide (DMSO), potassium persulfate, and all reagents were purchased from Sigma (St. Louis, MO, USA), Fluka Chemie (Buchs, Switzerland), and Merck (Nottingham, UK).

### 2.2. Plant Materials

The aerial parts of *T. capitata* were collected from Zaghouan region (north Tunisia) in June 2010. The samples species were identified and confirmed by a specialist in botany. The freshly cut plants were sorted out and dried in the shade at ambient temperature for two weeks. Dried samples were grounded into powder, packed in paper bags, and stored in the dark in a dry place.

### 2.3. Preparation of the Essential Oils

The dried powder aerial parts of plant were submitted to hydrodistillation process in a clevenger-type apparatus for 3 hours according to the method recommended in the current European Pharmacopoeia 6.0 in 2008 [23]. The EO collected was then dried over anhydrous sodium sulphate (Na_2_SO_4_), filtered, and stored at 4°C in the dark for further use.

### 2.4. Chemical Composition of Essential Oil

#### 2.4.1. Apparatus

GC-MS analysis of the essential oil was carried out with Hewlett Packard 7890 A GC equipped with a 5975 mass selective detector and an HP-5 MS capillary column (30 m × 0.25 mm id, film thickness 0.25 *μ*m). For GC/MS detection, the ion source was set to 230°C with electron ionization energy of 70 eV. Scanning range was varied from 40 to 550 atomic mass units (amu). Helium was used as the carrier gas at a flow rate of 0.8 mL/min. One *μ*L of diluted oil in hexane (1/100, v/v) was injected manually in splitless mode. The oven program temperature was programmed from 60°C to 250°C with a rate of 4°C/min and then held constant for 5 min.

#### 2.4.2. Qualitative and Quantitative Analyses of EO

The identification of the chemical compounds of EO was based on mass spectral library (Wiley 275.L, 8th edition) and/or with standards when available and confirmed by comparison of their GC retention indices either with those of authentic standards injected under the same chromatographic conditions or with data published in the literature, as described by Adams in 2007 [24].

### 2.5. Quantification of Total Antioxidant Activity

The literature outlines different approaches for the determination of the antioxidant activities of the plant extracts. Therefore, generally different methodological approaches lead to scattered results, which are hardly comparable and sometimes conflicting [25, 26]. For that reason, we combined two complementary techniques, based on DPPH and ABTS free radical-scavenging activity.

#### 2.5.1. DPPH Radical-Scavenging Assay

Radical-scavenging activity (RSA) of plant extracts against stable DPPH was determined by spectrophotometry. EOs extracts at different concentrations (0.1; 0.25; 0.5; 1; 5; 10; 50; 100; 200 *μ*g/mL) were mixed with the same volume of 0.2 mM methanolic DPPH solution. Samples were kept in the dark for 30 min at room temperature, and absorption-decrease was measured. Absorption of negative control containing the same amount of methanol and DPPH solution was prepared and measured in the same time. The experiment was carried out in triplicate. RSA of extracts was measured by the method described by Brand-Williams et al. in 1995 [27] but slightly modified as shown below:

(1) $$\begin{array}{c} \text{Inhibition}\;\%=\left[\frac{AB-AA}{AB}\right]\times 100, \end{array}$$

where *AB* is *AB* absorption of blank sample at *t* = 0 min and *AA* is the tested sample absorption at *t* = 30 min.

The antioxidant activity was also expressed as IC_50_, which was defined as effective concentration of the sample (in *μ*g/mL) at which 50% of DPPH radicals are scavenged. BHT and ascorbic acid were used as positive control. Each assay was repeated 3 times. The average result and standard deviation were reported.

#### 2.5.2. ABTS Activity

ABTS radical-scavenging activity of EOs was determined according to Re et al. in 1999 [28]. The ABTS solution was diluted with methanol, to absorbance of 0.7 at 734 nm. After the addition of 950 *μ*L of diluted ABTS solution to 50 *μ*L of plant EOs, the mixture was incubated at 37°C for 10 min, and then the absorbance was measured at 734 nm. Tests were carried out in triplicate. Butylated hydroxytoluene (BHT) and ascorbic acid (*AA*) were used as positive controls.

The ABTS radical-scavenging activity of the sample was calculated by the following equation:

(2) $$\begin{array}{c} \text{Inhibition}\;\left(\%\right)=\left[\frac{\text{Abs}\;\text{Control}-\text{Abs}\;\text{Sample}}{\text{Abs}\;\text{Control}}\right]*100, \end{array}$$

where Abs control is the absorbance of ABTS radical + methanol and Abs sample is the absorbance of ABTS radical + sample (EO/standard).

Sample concentration providing 50% inhibition (IC_50_) was obtained plotting the inhibition percentage against sample concentrations.

### 2.6. Antimicrobial Screening

#### 2.6.1. Microorganisms and Growth Conditions

The EO was tested against a large panel of microorganisms. Bacteria were obtained from international culture collections ATCC and the local culture collection of *Pasteur Institute of Tunis*. They included 8 Gram-positive bacteria and 16 Gram-negative bacteria (Table 1). The bacterial strains were cultivated in Luria Bertani Medium (LB) (Oxoid Ltd., UK) at 37°C except for *Bacillus* species, which were incubated at 30°C. Working cultures were prepared by inoculating a loopful of each test bacteria in 5 mL of Luria Bertani Medium (LB) (Oxoid Ltd., UK) and incubated at 37°C for 18 hours.

#### 2.6.2. Disc-Diffusion Method

The paper disc-diffusion method was employed for the determination of EO antimicrobial activity [29]. Briefly, suspension in LB of the tested microorganism (0.1 mL of 10^7^-10^8^ cells per mL) was spread on the solid LB media plates. Paper discs (9 mm in diameter) were individually impregnated with 12 *μ*L of the oil and then placed on the inoculated plates. We did not use the DMSO to facilitate the solubilization of EO in LB-Agar. However, in order to accelerate diffusion of the essential oil, plates were placed at 4°C for 2 hours and were then incubated at 37°C for 24 hours. The diameters of the inhibition zones were measured in millimeters. All tests were performed in duplicate and repeated three times. Streptomycin B (15 *μ*g/mL) and chloramphenicol (30 *μ*g/mL) were used as positive controls.

#### 2.6.3. Determination of the Minimum Inhibitory Concentration

The Minimal Inhibitory Concentrations (MICs) of the EO against the tested microorganisms were determined by the broth microdilution method [30]. All tests were performed in LB, supplemented with DMSO (the highest final concentration 0.1%). Microbial strains were cultured overnight at 37°C and were suspended in LB medium to give a final density of 5 × 10^5^ CFU/mL, which was confirmed by viable counts. Geometric dilutions ranging from 0.039 mg/mL to 20 mg/mL of the EOs were prepared in 96-well microtiter plate (Iwaki brand, Asahi Techno Glass, Japan), including one growth control (LB+DMSO), and one sterility control (LB+DMSO+ test oil). Plates were subsequently incubated under normal atmospheric conditions at 37°C for 24 hours and under vigorous agitation. The wells were then examined for evidence of growth indicated by the presence of white “pellets” on their bottoms. MICs values were determined as the lowest EO concentration that inhibited visible growth of the tested microorganism. The negative controls were set up with DMSO in amounts corresponding to the highest quantity present in the test solution (0.1%). The tests were performed three times.

### 2.7. Inhibitory Effect of the EO against Listeria Inoculated in Minced Beef Meat

The *in situ* efficacy of the EO was evaluated against *L. monocytogenes* in a minced beef meat model according to the procedure described by Careaga et al. in 2003 [31] but with a slight modification.

#### 2.7.1. Preparation of Meat Beef

Freshly postrigor lean beef muscles were obtained from a slaughter house in Tunis,Tunisia. Each piece was immersed in boiling water for 5 min, in order to reduce the number of the microorganisms attached to the beef muscle surface. The cooked surface of the muscle was eliminated with sterile knives under aseptic conditions.

#### 2.7.2. Treatment of Minced Beef

Prior to minced beef contamination with *Listeria monocytogenes* and the addition of EO, beef muscles were also examined for any contamination by bacteria (aerobic psychrotrophic flora) and the tested pathogens (results not shown). In order to evaluate the antimicrobial activity of *T. capitata* EO in a meat beef sample, the pieces of meat prepared as above were minced in a sterile grinder, and portions of 25 ± 0.1 g were put in high-density polyethylene bags. The meat samples were inoculated with *L. monocytogenes in concentration of* 10^5^ CFU/g of meat and mixed homogeneously for 3 min at room temperature to ensure proper distribution of the pathogen. Following homogenization, the *T. capitata* EO was dissolved in 10% DMSO and was subsequently added at different concentrations (0.02; 0.06; 0.1; 1; 1.5; 2 and 3 % (v/w)) to the inoculated samples. To obtain uniform distribution of the added compounds, treated meat samples were then homogenized by means of a Stomacher 400 Seward (London,UK) used at a normal speed for 5 min. All bags containing these samples of meat were stored at 7°C and examined at 0, 3, 6, 9, 12, and 15 days of storage for *L. monocytogenes *enumeration. The untreated samples (controls) were added to sterile water (instead of EO), inoculated with the test bacteria, and stored under the same conditions as the tested samples. Three replicates of each experiment were performed in all cases.

#### 2.7.3. Bacterial Enumeration

A microbiological analysis was performed on the meat, with the aim to assess quantitatively and qualitatively the background microflora. *L. monocytogenes* count was done adding 250 mL of Muller-Hinton broth to the 25 g in the polyethylene bag. The samples were homogenized for one min and incubated at 37°C for 6 hours. From this pre-enrichment, the *L. monocytogenes* was determined by the plate colony count technique. After serial 10-fold dilution with physiological saline solution, 100 *μ*L of each sample was spread onto surfaces of the Muller- Hinton agar medium followed by incubation at 37°C for 24 hours. Sterile saline water was added to the untreated control, inoculated with the test bacteria instead of *T. capitata* EO stored under the same conditions as the other samples.

### 2.8. Statistical Analysis

The inhibitory concentration 50% (IC50 values) for antioxidant activities was calculated by nonlinear regression analysis using the Graphpad Prism version 5.0. The dose-response curve was obtained by plotting the percentage of inhibition versus the concentrations. Correlations between inhibition activity and EO concentration were evaluated using Spearman's correlation test [32]. Statistical significance of the differences between the treated and the control sample means was evaluated by Welch 2-sample *t*-test. Repeated ANOVA test [33] was used to check overall difference in activity tendency and EO concentration effect. A *P* value <0.05 was considered to imply significance; however, corrections for multiple testing were carried out when necessary. All computations were performed using The R software 2.11 version (http://www.r-project.org/).

## 3. Results and Discussion

### 3.1. Chemical Composition of the Extracted Essential Oils

Table 2 shows the chemical constituents, their relative percentage of the total chromatogram area and Kovats index of *T. capitata* EO.

GC-MS analysis of the volatile constituents of the EO allowed the identification of 19 compounds representing 98.97% of the total oil. Carvacrol was the major one with 88.98%. The other identified components were minor. These results are in line with those reported by Napoli et al. [34]. The chemical composition of this EO showed that it is rich in oxygen containing monoterpenes (94.98%). Monoterpene hydrocarbons or both sesquiterpene and oxygen containing sesquiterpene were represented at about 2% each. This wealth of oxygen-containing monoterpenes (OM), especially carvacrol, can enhance the value of this EO as an active natural product. The major product carvacrol was described as a strong antibacterial molecule [9, 11] and it is now considered one of the products singled out for their pharmacological effects. These results are in accordance with previous studies [35, 36], which demonstrated that carvacrol was the main compound of *T. capitata* oils with 75% and 65.8%, respectively.

On the other hand, there are many reports on the chemical composition of other oils isolated from the plants belonging to the genus of *thymus*. Tomaino et al. in 2005 [37] reported that the major constituents of thyme EO were carvacrol, thymol, and *p-*cymene and they can reach the following percentages: 48.9%, 45.3%, and 26.19%, respectively, while Jaafari et al. in 2007 [38] found that these same constituents are the main components in thyme EO from Morocco and can hit the following percentages: 85%, 42%, and 23%, respectively. These variations in the composition of the EO could be due to factors such as plant age, plant part, development stage, the geographical localization, harvesting period, temperature, and environmental factors prevailing in the Mediterranean regions and principally by chemotype since they influence the plant biosynthetic pathways and consequently, the relative proportion of the main characteristic compounds [39].

### 3.2. Antioxidant Activity

**Figure 1:**
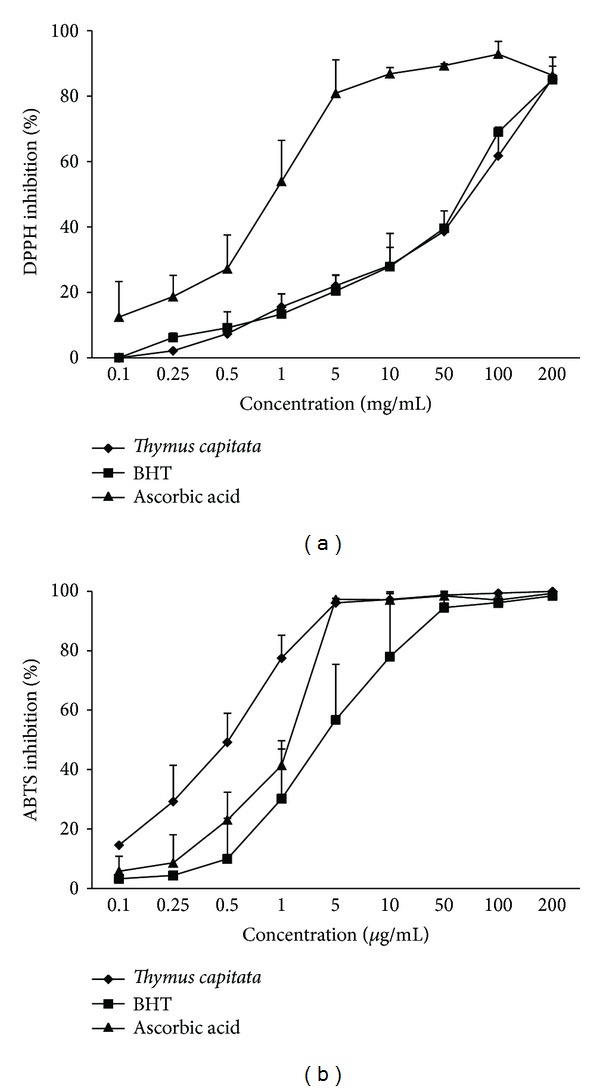
The antioxidant activities of *Thymus capitata* essential oil as determined by DPPH (a) and ABTS (b) free radical-scavenging activity. The absorbance values were converted to scavenging effects (%) and data plotted as the means of replicate scavenging effect (%) values. (Results are expressed as means ± standard deviation of three measurements.)

Two complementary colorimetric methods, namely the DPPH and ABTS assays are compared to the reference standards butylated hydroxyl toluene (BHT) and ascorbic acid (AA), and the results are presented in Figure 1. The DPPH and the ABTS radicals are the two most widely used and stable chromogen compounds to measure the antioxidant activity of biological material [40]. In addition, the model of the DPPH radical-scavenging and ABTS radical cation decolorization assay can be used to evaluate the antioxidant activities in a relatively short time compared with other methods [41, 42]. In the present study, the capacity of the EO to scavenge the free radicals DPPH^•^ and ABTS^+•^ and their reducing power was determined on the basis of their concentration providing 50% inhibition (IC_50_) and the lower IC_50_ value reflects high radical-scavenging activity [43].

#### 3.2.1. DPPH Free Radical-Scavenging Activity

The effect of antioxidant on DPPH radical-scavenging was conceived to their hydrogen-donating ability [44]. DPPH is a stable free radical that accepts on electron or hydrogen radical to become a stable diamagnetic molecule [43].

From the analysis of Figure 1(a), we can conclude that the radical-scavenging activity of the EO and positive controls increased with increasing concentration (Spearman correlations *r* = 0.856 with *P* values <0.0001). Furthermore, the results obtained in this study indicated that the *T. capitata* EO exhibited a high DPPH radical-scavenging activity and its percentage inhibition reached 85.44 ± 1.06% at a concentration of 200 *μ*g/mL. The graph (Figure 1(a)) showed that the radical-scavenging activity of *T. capitata* EO was 44.16 ± 0.809 *μ*g/mL, which appeared lower than of synthetic antioxidants BHT and ascorbic acid, with values of IC_50_ = 39.97 ± 1.64 *μ*g/mL and 1.136 ± 0.305 *μ*g/mL, respectively.

#### 3.2.2. ABTS Free Radical-Scavenging Activity

Similar to DPPH, the decolorization of ABTS radical reflects the capacity of an antioxidant species to donate electron or hydrogen atoms to inactivate this radical cation [45]. The ABTS results were in good agreement with DPPH method that the scavenging activity of the EO was increased with the increasing concentration (Spearman correlations *r* = 0.89 with *P* values <0.0001). From the analysis of Figure 1(b), we can conclude that the *T. capitata* EO exhibited higher ABTS radical-scavenging activity (99.98 ± 0.01%), which was comparable to that of BHT (98.46 ± 0.95%) and ascorbic acid (99.33 ± 0.59%) for the same concentration 200 *μ*g/mL. These findings were confirmed by calculating the IC_50_ values for the *T. capitata* EO (IC_50_ = 0.463 ± 0.122 *μ*g/mL), which was found to be significantly (*P* < 0,05) better than that of BHT (IC_50_ = 3.204 ± 3.541 *μ*g/mL) and ascorbic acid (IC_50_ = 1.126 ± 0.19 *μ*g/mL). These results are in agreement with previous studies [46, 47], which showed that greater antioxidant potential of several *Thymus* species EOs could be related to the nature of phenolic compounds and their hydrogen ability. Besides, it could be ascribed to the oxygenated types of compounds, such as carvacrol and thymol [26, 48]. Moreover, the activities of EOs of *Thymus* species depend on several structural features of the molecules and are primarily attributed to the high reactivity of hydroxyl group substituent [49].

Scavenging the ABTS radical by the *T. capitata* EO was found to be much higher than that of DPPH radical. These differences can be explained by the mechanism of the involved reaction. The ABTS radical reactions involve electron transfer and take place at a much faster rate compared to DPPH radicals [50]. Furthermore, various factors like stereoselectivity of the radicals or the solubility of the tested sample in different testing systems and functional groups present in the bioactive compounds have been reported to affect the capacity of the sample to react and quench different radicals [51]. Wang et al. in 1998 [52] showed that some compounds which have ABTS^+^ scavenging activity may not show DPPH scavenging activity.

### 3.3. Antimicrobial Activity

In the present study, the *in vitro* antimicrobial activities of *T. capitata *EO against the studied microorganisms were qualitatively and quantitatively assessed by the presence or absence of inhibition zones and MIC values, respectively (Table 3). The results obtained from the disc- diffusion method indicated that EO exerted a strong antibacterial activity against all tested strains. Results were comparable to those of the antibiotics (chloramphenicol and streptomycin), used as positive controls. The size of the inhibition zone of *T. capitata* EO varied from 15 to 80 mm, while the inhibition zones of the chloramphenicol and streptomycin ranged from 18–27 mm to 12–22 mm, respectively.

Referring to the large inhibition zones observed with disk-diffusion method for *T. capitata* EO, the MIC values were determined by the microdilution broth assay (Table 3). The results of the MIC values against tested Gram-positive and Gram-negative bacteria varied from 0.32 to 5 mg/mL and from 0.63 to 20 mg/mL, respectively. We found that the antibacterial activity of the EO depends on its concentration and the tested bacteria strain. Interestingly, we have found that *Staphylococcus aureus* ATCC 6538 is the most sensitive tested microorganism, with the lowest MIC value (0.32 mg/mL), and it was closely followed by *Bacillus cereus* ATCC 11768. This antimicrobial spectrum obtained with the EO of *T. capitata* is comparable in most cases to the one reported by Bounatirou et al. in 2007 [9]. In addition, *Vibrio cholerae* (clinical isolate) is the most sensitive Gram-negative bacteria with the lowest MIC value (0.63 mg/mL). Our results confirmed that Gram-positive bacteria were more susceptible to the antimicrobial properties of EO than Gram-negative ones. These differences could be attributed in part to the great complexity of the double membrane-containing cell envelope in Gram-negative bacteria compared to the single membrane structure of the positive ones [53, 54]. These differences may be attributed also to the presence of the lipopolysaccharides in the outer membrane of the Gram-negative bacteria, which make them inherently resistant to external agents, such as hydrophilic dyes, antibiotics, detergents, and lipophilic compounds [14, 55]. However, the ability of EOs to disrupt the permeability barrier of cell membrane structures and the accompanying loss of chemiosmotic control is the most likely reason for its lethal action [56]. The EOs can coagulate the cytoplasm and damage lipids and proteins [3]. Their mechanism of action would be similar to other phenolics, that is, the disturbance of the proton motive force, electron flow, active transport, and coagulation of cell contents. Instead, enzymes such as ATPases are known to be located in the cytoplasmic membrane and to be bordered by lipid molecules [3, 13].

Generally, antimicrobial activities of the EOs are difficult to correlate with a specific compound due to their complexity and variability; nevertheless, some investigators reported that there is a relationship between the chemical composition of the most abundant components in the EO and the antimicrobial activity [57, 58]. In the present study, carvacrol was the main component of *T. capitata* EO. It has been reported to be biocidal, resulting in bacterial membrane perturbations that lead to leakage of intracellular ATP and potassium ions and ultimately cell death [59, 60]. Previous studies [61] mentioned that carvacrol at concentrations of 0.5% and 1% shows antibacterial activity against *Shigella sonnei* and *Shigella flexneri*. Besides, it has been reported that carvacrol causes perturbation in the bacterial membrane and thus potentially can exert antibacterial activity also at intracellular sites [60, 62]. These results are in accordance with the earlier findings [12, 54] that showed that *Thymus* species' essential oils rich in carvacrol were demonstrated to be potent antimicrobial *in vitro*.

However, other constituents, such as terpinene and *p-*cymene have been shown to display relatively good activity due to their potential synergistic or antagonistic effects [10, 63].

### 3.4. The Effects of the EOs on *L. monocytogenes* Inoculated in Minced Beef Meat

**Figure 2:**
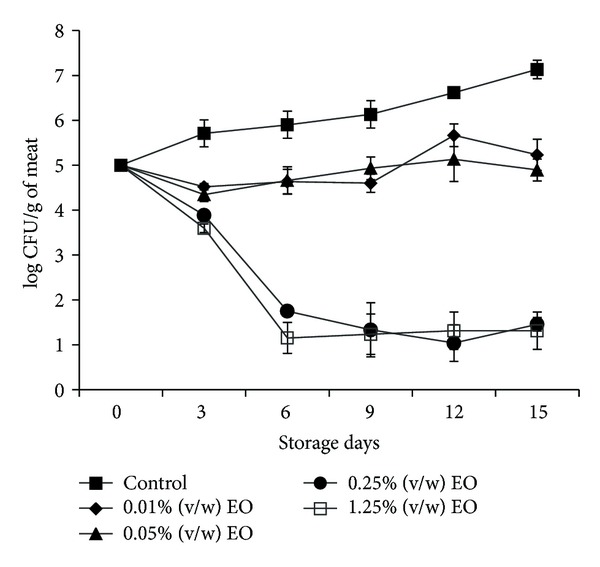
Time-related survival of *Listeria monocytogenes* at 7°C following treatment with increasing concentrations of *Thymus capitata* essential oil. Bacteria were supplemented in minced beef meat samples at 10^5^ CFU/g of meat. Values are the average of three individual replicates.

In this part of our work, we studied *in vivo* the anti-*Listeria* activity of different concentration of *T. capitata* EO when inoculated in minced beef meat, as well as the effect of EO on the extension of shelf life and the preservation of the freshness of meats. It is well known that not all microbiologists demonstrated that decontamination of meat is required or even desirable. It has been argued by Jay in 1996 [64] that high levels of indigenous nonpathogenic microorganisms may have a protective effect on meat and its products, by out-competing the pathogens. Despite this fact, our samples were decontaminated in order to reduce the number of factors involved in the microorganisms' growth in such food model and to avoid interferences of colonies on plating agar. The bacteria count, which is related to survival time of *L. monocytogenes* in our processed food model following treatment with various concentrations (0.01; 0.05; 0.25, and 1.25% (v/w)) of *T. capitata* EO, was presented in Figure 2. Results showed that the initially recorded population of *Listeria monocytogenes* in untreated samples (control) increased approximately from 5log CFU/g to 7.13 log CFU/g during 15 days of storage. However, data from each of the four preparations showed a gradual decrease in the bacteria count with the increasing EO concentration. It appears that the used concentrations are higher than those applied for the *in vitro* tests. This cannot be misleading, because it is well established that intrinsic factors such as composition (e.g., proteins, fat) as well as extrinsic factors (temperature, oxygen limitation) of the food affect the behavior of bacteria in food ecosystems and may act synergistically with preservatives such as antimicrobial agents [32]. Indeed, food components, such as proteins and fat, are known to bind and/or solubilized phenolic compounds, reducing their availability for antimicrobial activity. Furthermore, it has been reported by many authors that antimicrobial activity of spice is lower in food systems than in microbiological media [65].

Indeed, a reduction of 4×log/g in the level of *L. monocytogenes* was recorded in 3 days of storage with a concentration of 0.25 or 1.25% (v/w) of *T. capitata* EO, compared to the control (not treated), and those treated either with a concentration of 0.01 or 0.05% (v/w) of *T. capitata* EO. The differences in the values were statistically significant (*P* values <0.001). Thus, at the end of experimentation (15 days of storage), bacteria count in minced beef treated with a concentration of 0.25 and 1.25% (v/w) of *T. capitata* EO decreased and reached 1.45 and 1.13×log CFU/g, respectively. However, we did not notice immediate lethal (bactericidal) effects on *L. monocytogenes* when *T. capitata* EO was applied as described by other studies [66, 67], but we observed a strong inhibitory activity against *L. monocytogenes*. These discrepancies between our results and others that found full lethal effects can be explained by the fact that the activity depends on the type, composition and the concentration of the EO, the strain, and the dose of target microorganism inoculated in the meat. In this study, we used high inoculum (10^5^ CFU/g) before treating mince beef compared to low inoculum (10^3^ CFU/g) used by Hsouna et al. in 2011 [67]. Taken together, these results demonstrated that EOs derived from *T. capitata* have a great potential in terms of activity against the tested strains of *L. monocytogenes*. Thus, the dose-related inhibitory activity suggests the possibility of using this product as meat preservative. In agreement with our findings, Djenane et al. in 2011 [14] showed a high decrease of bacteria load when minced beef is treated with *Pistacia lentiscus* and *Satureja montana* EOs against *Listeria monocytogenes* CECT 935.

These results are in accordance with previous studies reveling that thyme EO significantly reduced viable counts of *Listeria monocytogenes* in Russian-type salad during one-week storage at 10°C when combined with Enterocin AS-48 (30–60 *μ*g/g) [68] and exhibited a reduction about 0.25% of initial populations of *L. monocytogenes* in minced pork by 2 and 2.3×log CFU/g after 8 days of storage at 4°C and 8°C [69]. In fact, the potent antimicrobial activities of *T. capitata* EO observed in this study can be attributed to the presence of high concentration of carvacrol, which has a well-documented antibacterial potential [14].

## 4. Conclusion

In conclusion, this study focused on the correlation between the chemical concentration and the effectiveness of *T. capitata* EO as an antioxidant and antimicrobial. The results of this work show that *T. capitata* EO can exhibit strong antioxidant and antimicrobial activity, probably due to its particular chemical composition, mainly the high amounts of carvacrol. In the second part, *L. monocytogenes* populations in minced beef treated with essential oil were significantly lower than those in control samples throughout the storage period. The application of 0.25 or 1% EOs (v/w) of *T. capitata* EO to minced beef coupled with low temperature storage can reduce the potential of *L. monocytogenes* contamination. So, this EO can be used for the preservation of meats against *L. monocytogenes* and for increasing their shelf life. All results obtained herein suggest that the *T. capitata* EO exhibited a bioprotector effect and therefore it could be used in many biotechnological fields as a natural preservative ingredient of food and/or pharmaceutical industries.

## Figures and Tables

**Table 1:** Bacteria strains used.

Gram-negative bacteria	Gram-positive bacteria
*Escherichia coli* ATCC 25922	*Enterococcus faecalis *ATCC 11700
*Enterobacter cloacae* ATCC 13097	*Listeria monocytogenes *ATCC 19118
*Proteus mirabilis* ATCC 29906	*Staphylococcus aureus *ATCC 6538
*Pseudomonas aeruginosa* ATCC 27853	*Staphylococcus aureus *ATCC 25923
*Pseudomonas aeruginosa* ATCC 9027	*Staphylococcus aureus *ATCC 6538
*Salmonella enteritidis* ATCC 502	*Streptococcus pyogenes *ATCC 12344
*Salmonella salamae* ATCC 6633	*Bacillus cereus *ATCC 11778
*Salmonella typhimurium* ATCC 14028	*Bacillus cereus* (food isolate)
*Shigella flexneri *ATCC 29903	*Bacillus subtilis* (food isolate)
*Yersinia enterocolitica* ATCC 23715	
*Klebsiella oxytoca* (clinical isolate)	
*Morganella morganii* (clinical isolate)	
*Pseudomonas aeruginosa* (clinical isolate)	
*Salmonella anatum* (food isolate)	
*Shigella sonnei *(clinical isolate)	
*Vibrio cholerae *(clinical isolate)	

**Table 2:** Chemical composition of the essential oil isolated from the aerial parts of *Thymus capitata* from Zaghouan region (Tunisia).

Compounds	Retention time	%^a^	RI^b^	Method of identification^c^
1-Octen-3-ol	6.434	0.25	987.179	RI, MS
Beta-myrcene	6.749	0.11	1020.159	RI, MS
*α*-Terpinen	7.481	0.15	1018.808	RI, MS
*p-*Cymene	7.699	1.14	1026.317	RI, MS
*γ*-Terpinene	8.677	0.40	1060.006	RI, MS
Sabinene hydrate	8.946	0.09	1069.27	RI, MS
Linalol	9.902	1.57	1101.99	RI, MS
Borneol	12.070	1.06	1169.64	RI, MS
Terpinen-4-ol	12.431	1.41	1180.90	RI, MS
*α*-Terpineol	12.866	0.29	1194.47	RI, MS
Trans-dihydrocarvone	13.066	0.11	1200.07	RI, MS
Beta-citral	14.474	0.24	1243.84	RI, MS
Carvone	14.600	0.18	1247.70	RI, MS
Citral	15.481	0.33	1274.69	RI, MS
Thymol	16.202	0.51	1296.78	RI, MS
**Carvacrol**	16.688	**88.98**	1311.93	RI, MS
Caryophyllene	20.264	0.63	1425	RI, MS
Caryophyllene epoxide	25.191	1.08	1589.88	RI, MS
Dodecyl acrylate	28.144	0.44	1695.47	RI, MS
Total		**98.97**		

**(2)** Compounds are listed according to their elution on HP-5MS capillary column.

^a^Peak area of essential oil components.

^b^Kovats retention indices relative to C_9_–C_20_ *n*-alkanes on the HP-5MS capillary column.

^c^Components were identified based on their KI on HP-5MS capillary column and GC-MS data.

**Table 3:** Antibacterial activity of essential oil from *Thymus capitata*, using paper disc-diffusion method and microdilution test.

Strains	Disc-diffusion method (DD)			MIC
	*Thymbra capitata *(L.)	Antibiotics		*Thymbra capitata *(L.)
		a	b	
*Pseudomonas aeruginosa* ATCC 27853	23	21	12	10
*Pseudomonas aeruginosa* ATCC 9027	15	19	13	20
*Pseudomonas aeruginosa* (clinical isolate)	17	22	16	20
*Escherichia coli* ATCC 25922	70	NA	12	2.5
*Enterococcus faecalis*ATCC 11700	60	20	14	2.5
*Enterobacter cloacae* ATCC 13097	80	18	13	5
*Salmonella typhimurium* ATCC 14028	50	22	15	2.5
*Salmonella enteritidis* ATCC 502	80	21	13	5
*Salmonella salamae* ATCC 6633	75	22	15	5
*Salmonella anatum* (food isolate)	80	20	18	2.5
*Shigella flexneri *ATCC 29903	80	18	15	2.5
*Shigella sonnei *(clinical isolate)	80	20	14	1.25
*Staphylococcus aureus *ATCC 2592	20	20	22	5
*Staphylococcus aureus* ATCC 6538	75	23	NT	0.32
*Streptococcus pyogenes* ATCC 12344	75	21	NT	2.5
*Listeria monocytogenes* ATCC 19118	70	23	16	5
*Morganella morganii* (clinical isolate)	75	NT	NT	1.25
*Klebsiella oxytoca* (clinical isolate)	70	21	15	2.5
*Vibrio cholerae *(clinical isolate)	80	NT	NT	0.63
*Yersinia enterocolitica* ATCC 23715	80	NT	NT	10
*Proteus mirabilis* ATCC 29906	45	NT	NT	5
*Bacillus cereus *ATCC 11768	50	20	16	0.63
*Bacillus cereus* (food isolate)	80	NA	NA	1.25
*Bacillus subtilis *(food isolate)	70	27	15	5

**(3)** Disc-diffusion method. Inhibition zone in diameter around the discs impregnated with 12 *μ*L of essential oil. The diameter (9 mm) of the disc is included.

MIC: minimal inhibitory concentration; values given as mg/mL for the essential oils.

a: Chloramphenicol (30 *μ*g/*μ*L); b: streptomycin B (10 *μ*g/*μ*L); NT: not tested; NA: not active.

## Abbreviations

EOs: Essential oils
*T. capitata*: *Thymus capitata*
GC-MS: gas chromatography, mass-spectrometry.
